# Modulation of mitochondrial nucleoid structure during aging and by mtDNA content in *Drosophila*

**DOI:** 10.1242/bio.058553

**Published:** 2021-06-28

**Authors:** Li-jie Wang, Tian Hsu, Hsiang-ling Lin, Chi-yu Fu

**Affiliations:** Institute of Cellular and Organismic Biology, Academia Sinica, Taipei 115, Taiwan

**Keywords:** Mitochondrial DNA, Mitochondrial nucleoid, Transcription factor A (TFAM), Mitochondrial RNA polymerase (mtRNAPol), Mitochondrial transcription factor B2 (mtTFB2)

## Abstract

Mitochondrial DNA (mtDNA) encodes gene products that are essential for oxidative phosphorylation. They organize as higher order nucleoid structures (mtNucleoids) that were shown to be critical for the maintenance of mtDNA stability and integrity. While mtNucleoid structures are associated with cellular health, how they change *in situ* under physiological maturation and aging requires further investigation. In this study, we investigated the mtNucleoid assembly at an ultrastructural level *in situ* using the TFAM-Apex2 *Drosophila* model. We found that smaller and more compact TFAM-nucleoids are populated in the mitochondria of indirect flight muscle of aged flies. Furthermore, mtDNA transcription and replication were cross-regulated in the *mtTFB2*-knockdown flies as in the *mtRNAPol*-knockdown flies that resulted in reductions in mtDNA copy numbers and nucleoid-associated TFAM. Overall, our study reveals that the modulation of TFAM-nucleoid structure under physiological aging, which is critically regulated by mtDNA content.

## Abstract (precis)

**Summary:** The TFAM-nucleoid structure is critically regulated by mtDNA content and changes during aging. Mitochondrial transcription factor B2 plays a role in mtDNA replication.

## INTRODUCTION

Mitochondria participate in a wide range of metabolic processes and cellular functions, such as metabolism, lipid synthesis, calcium homeostasis, and cell death (Nunnari and Suomalainen, 2012). Even though the majority of mitochondrial proteins are encoded by the nuclear genome and imported from the cytoplasm, the principal function of oxidative phosphorylation is carried out by the proteins encoded by both the nuclear genome and mitochondrial DNA (mtDNA) (Kang et al., 2018; Nicholls and Gustafsson, 2018). Mitochondria themselves contain multiple copies of a small double-stranded circular DNA (mtDNA), which encodes several core subunits of electron transport chain (ETC) complexes, as well as rRNAs and tRNAs to support protein synthesis (Kang et al., 2018; Nicholls and Gustafsson, 2018). In response to cellular physiological conditions, the replication and transcription of mtDNA are coordinated with the expression of nuclear DNA-encoded mitochondrial genes to alter mitochondrial mass and activity (Bogenhagen et al., 2008; Kang et al., 2018; Nicholls and Gustafsson, 2018). Based on the essential role of mtDNA in metabolism, its stability and integrity are thought to participate in aging and various diseases, including neurodegenerative diseases, cancers, and diabetes (Kang et al., 2018; Nicholls and Gustafsson, 2018).

The mtDNA molecule is organized in a higher order structure, where it is packaged with a variety of proteins. This nucleoid-like structure (mtNucleoid) may either be long-lived or temporary and plays an essential role in regulating mitochondrial metabolism (Lee and Han, 2017). TFAM is a major structural constituent of the mtNucleoid and interacts with mtDNA independent of the nucleotide sequence (Lee and Han, 2017). It is a key regulator of nucleoid compaction and mtDNA stability (Farge et al., 2014; Kukat et al., 2015; Ngo et al., 2014).

While compromised mtDNA stability and integrity are associated with various pathological diseases, it is less known about how mtNucleoids are maintained in organisms under physiological maturation and aging. In this study, we investigated how mtNucleoid packaging was modulated during the *Drosophila* adult lifespan. To do so, we performed *in situ* ascorbate peroxidase (Apex2) staining to visualize mtNucleoids at an ultrastructural level using a TFAM-Apex2 knock-in fly where the Apex2 tag was fused to the c-terminus of endogenous TFAM to minimize possible confounding effects on TFAM expression level. Since TFAM is a major nucleoid structural protein, it has been used as a marker to image mtNucleoids by electron microscopy (EM) or fluorescent microscopy (FM) (Brown et al., 2011; Han et al., 2017; Kopek et al., 2012; Kukat et al., 2011; Legros et al., 2004; McArthur et al., 2018). We found that in the mitochondria of the indirect flight muscles (IFMs), smaller and more compact TFAM-nucleoids are more populated in aged flies. We also investigated how knocking down mtDNA transcription in *Drosophila* affects the mtDNA and TFAM assemblies as well as the ultrastructure of mtNucleoids.

## RESULTS

### TFAM-Apex2 fly as a model to visualize mtNucleoids

Higher order mtNucleoid structure was shown to be important for mtDNA stability and may serve to regulate mtDNA transcription (Farge et al., 2014; Kukat et al., 2015; Ngo et al., 2014). To address how mtNucleoid packaging is modulated during physiological maturation and aging, we generated a TFAM-Apex2 knock-in fly, where the Apex2 tag is fused to the C-terminus of the endogenous TFAM gene (Fig. S1). We applied an *in situ* Apex2-EM staining method to visualize mtNucleoids in the context of ultrastructure. Besides its role in regulating transcription directly, TFAM is a major structural constituent of the mtNucleoid that exists predominantly in its DNA-bound form and packages mtDNA independent of the nucleotide sequence (Lee and Han, 2017). TFAM has been used extensively as a marker to image mtNucleoids by EM or FM (Brown et al., 2011; Han et al., 2017; Kopek et al., 2012; Kukat et al., 2011; McArthur et al., 2018).

**Fig. 1.**
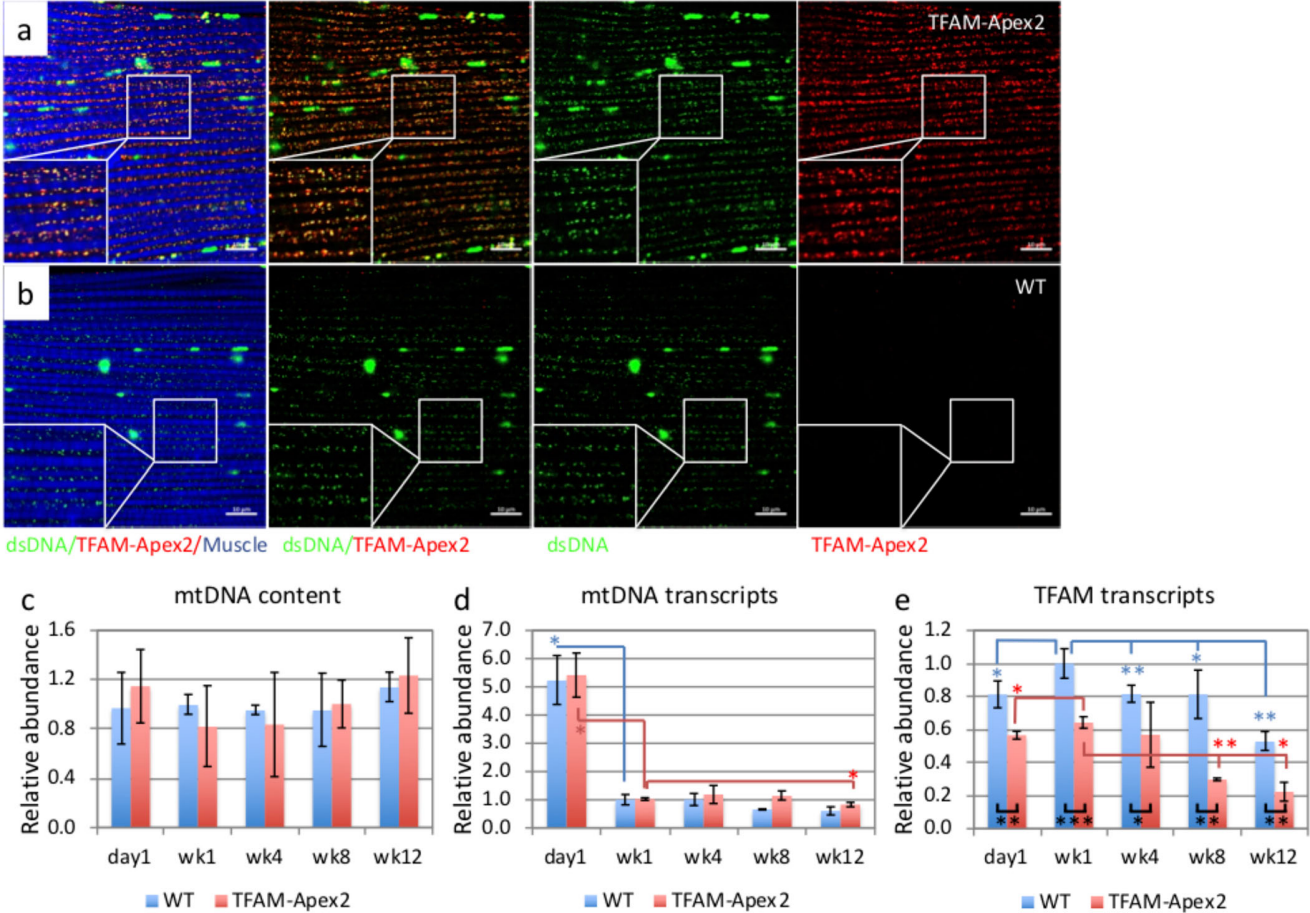
**TFAM-Apex2 fly as a model to visualize mtNucleoids.** Immunofluorescence staining of TFAM-Apex2 (A) and wild-type (B) fly IFM shows highly colocalized TFAM-Apex2 and mtDNA. Red: TFAM-Apex2; green: mtDNA; purple-blue: muscle fibers stained with phalloidin. Relative mtDNA content (C), mtDNA transcripts (D), and TFAM transcripts (E) of TFAM-Apex2 fly at day 1, week 1, week 4, week 8, and week 12 determined by qPCR and normalized to the level of wild-type week 1, respectively. The mean±s.d. of the triplicates was plotted and the *t*-test was performed. Statistically significant differences between indicated groups are marked with asterisks (*P<0.05; **P<0.01, compared to week 1).

The TFAM-Apex2 knockin fly is homozygous viable, which suggests that TFAM-Apex2 fusion protein can complement the absence of wild-type TFAM protein. Moreover, the association of TFAM-Apex2 with mtDNA was confirmed by immunofluorescence staining of IFM; TFAM-Apex2 protein detected by the antibody against its Flag-tag was highly colocalized with mtDNA detected by the antibody against dsDNA (Fig. 1A,B). The large foci stained by anti-dsDNA antibody are the cell nuclei. The TFAM-Apex2 fly had comparable mtDNA levels with the wild type as measured by qPCR, and no significant effects of age were found (Fig. 1C). The PCR products amplified by the primers specified in the methods were shown in Fig. S2. In both wild-type and TFAM-Apex2 fly, mtDNA transcripts were about fivefold higher on day 1, and there was a small but significant reduction at week 12, in comparison with week 1 (Fig. 1D). However, TFAM transcript levels of the TFAM-Apex2 fly were reduced significantly compared to the wild-type flies that we do not have clear reasoning for it (Fig. 1E). We then examined whether the TFAM-Apex2 knockin alters mitochondrial function significantly. TFAM-Apex2 flies showed comparable levels of ATP and ATP5A protein expression as well as a similar climbing ability to the wild types, suggesting that the TFAM-Apex2 flies have normal mitochondrial functions (Fig. S3a–c). Since the TFAM-Apex2 fusion did not exhibit a drastically altered function of TFAM, we considered the TFAM-Apex2 fly to be a relevant model for this study.

### Smaller TFAM-Nucleoids become more prevalent as flies age

**Fig. 2.**
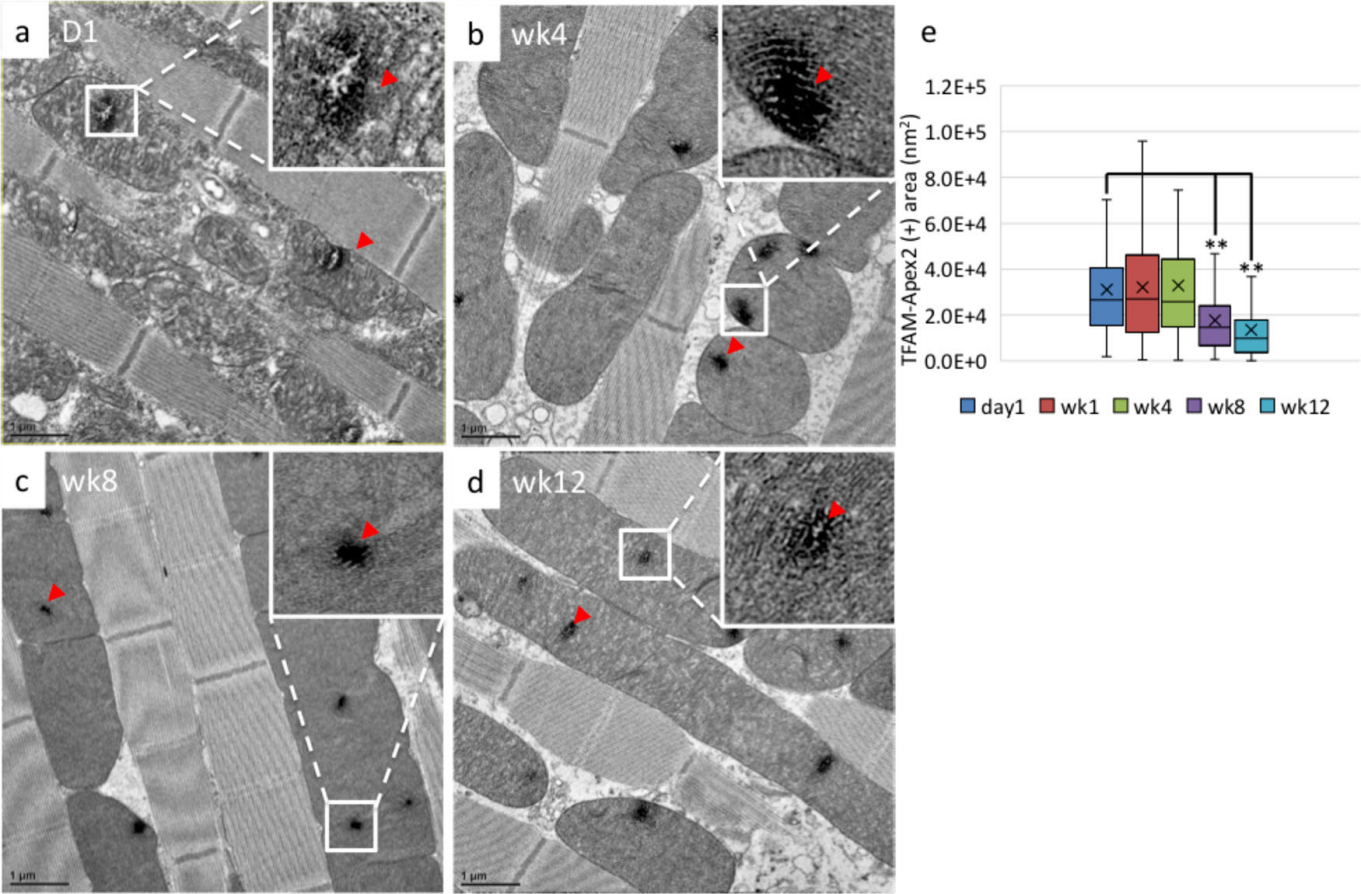
**Small mtNucleoids become more prevalent as flies age.** Apex2-staining EM images of the IFM of TFAM-Apex2 flies at day 1 (A), week 1 (B), week 4 (C), and week 12 (D). Red arrowheads indicate TFAM-Apex2 signals. (E) The size distributions of TFAM-Apex2 staining in flies of different ages were plotted in a box-and-whisker graph. The *t*-test was performed and statistically significant differences between indicated groups are marked with asterisks (*n*=106–164; **P<0.01 compared to the day 1). Mitochondria of week 8 and week 12 flies more frequently contained smaller mtNucleoids.

TFAM-Apex2 flies at various ages were sampled for Apex2-EM staining and thin-section EM analysis. Apex2, an ascorbate peroxidase, catalyzes the polymerization of DAB in the presence of hydrogen peroxide (H_2_O_2_) (Martell et al., 2012). The polymerized DAB then enhances EM contrast after osmium tetroxide staining, allowing us to track TFAM protein localization at the ultrastructural level as a surrogate marker of mtNucleoids. Micrographs of day 1 to week 12 fly IFM were analyzed, with TFAM-Apex2 signals appearing as darkly stained regions within the mitochondrial matrix (Fig. 2A–D). We analyzed the size distribution of TFAM-nucleoids from pools of 106–164 nucleoids at each age. The size distributions of TFAM-nucleoids on day 1, week 1, and week 4 appeared to be quite similar (Fig. 2E). At weeks 8 and 12, smaller TFAM-nucleoids were more common (Fig. 2E). However, the western blot did not detect significant differences in TFAM protein expression across ages (Fig. S3d). Therefore, we did not find an obvious correlation between the TFAM protein level and the frequency of smaller TFAM-nucleoids in older flies.

In summary, using the TFAM-Apex2 knockin fly model to visualize the mtNucleoid *in situ* revealed that the size distribution of TFAM-nucleoids changed as flies aged, with smaller nucleoids becoming more prevalent in older flies.

### The *mtRNAPol-* and *mtTFB2-* knockdown display reduced mtDNA content and mtDNA-associated TFAM in *Drosophila*

The replication and transcription of mtDNA were regulated by several dedicated mitochondrial regulators (Barshad et al., 2018; Nicholls and Gustafsson, 2018). Among them, the mitochondrial RNA polymerase (mtRNAPol), the mitochondrial transcription factor B2 (mtTFB2), and the mitochondrial transcription factor A (TFAM) were shown to be required for the initiation of mitochondrial transcription (Barshad et al., 2018; Falkenberg et al., 2002; Ojala et al., 1981). The mitochondrial transcription factor B1 (mtTFB1), on the other hand, was shown to be one order of magnitude less active in promoting transcription than mtTFB2. However, the methyltransferase activity of mtTFB1 on mitochondrial 12S rRNA modulates mitochondrial translation (Falkenberg et al., 2002; Matsushima et al., 2005; Metodiev et al., 2009). In addition, mtRNAPol was shown to prime the lagging-strand mtDNA synthesis *in vitro* (Fuste et al., 2010; Wanrooij et al., 2008). mtRNAPol regulates the switch between replication primer formation and gene expression on mammalian mtDNA (Kuhl et al., 2016). We investigated how knocking down *mtRNAPol* as well as *mtTFB2* affects mtDNA and TFAM-nucleoid packaging in *Drosophila* muscles.

TFAM-APEX2 was expressed in the IFM on *mtRNAPol-, mtTFB1-,* and *mtTFB2-RNAi* background was expressed in the IFM under the control of *Actin88F-GAL4.* Analyzing the transcript levels of *mtRNAPol*, *mtTFB1,* and *mtTFB2* by qPCR showed reductions of 64%, 67%, and 64%, respectively, in the knockdowns (Fig. S4a). Moreover, the mtDNA content and transcript levels in the *mtRNAPol-* and *mtTFB2-*knockdowns showed significant reductions, compared to the control (Fig. S3b). In comparison, the *mtTFB1-*knockdown flies had similar mtDNA content and transcript levels compared to the control (Fig. S4b).

**Fig. 3.**
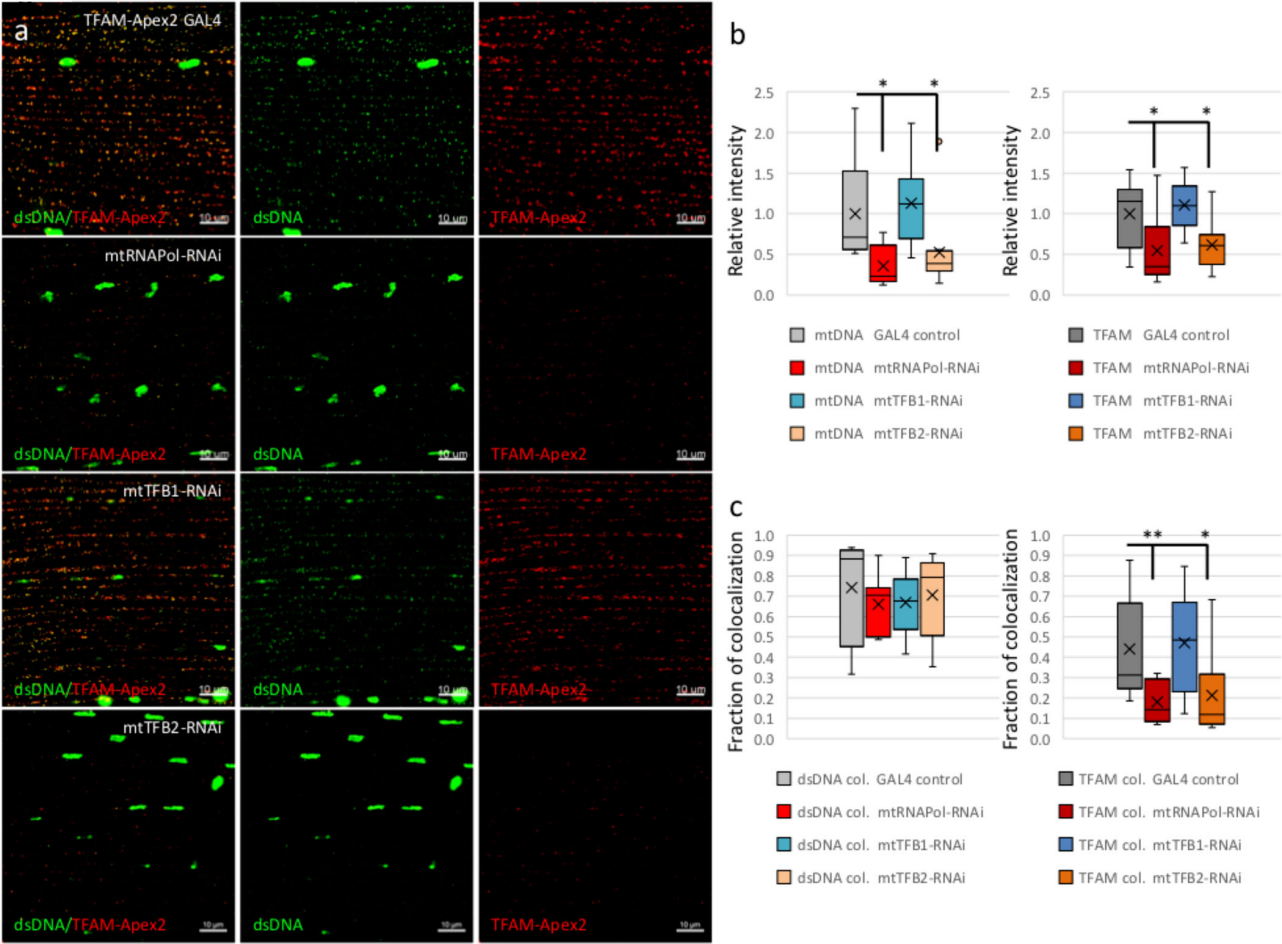
**The *mtRNAPol-* and *mtTFB2*-knockdown display reduced mtDNA content and increase the level of unbound TFAM.** (A) Immunofluorescence staining of the IFM of TFAM-Apex2 GAL4 control, TFAM-Apex2 under *mtRNAPol-*, *mtTFB1-*, and *mtTFB2-RNAi* flies. Red: TFAM-Apex2; green: dsDNA. (B) The analyses of immunofluorescence intensities of mtDNA and TFAM-Apex2 in TFAM-Apex2 GAL4 control and TFAM-Apex2 in *mtRNAPol-*, *mtTFB1-*, and *mtTFB2-RNAi* flies were plotted in box-and-whisker graphs. (C) The analyses of immunofluorescence colocalization of mtDNA with TFAM-Apex2 and TFAM-Apex2 with mtDNA of TFAM-Apex2 GAL4 control and TFAM-Apex2 in *mtRNAPol-*, *mtTFB1-*, and *mtTFB2-RNAi* flies were plotted in box-and-whisker graphs. The *t*-test was performed and statistically significant differences between indicated groups are marked with asterisks (*n*=9–12 volumes of 84.2×84.2×5 μm^3^ were analyzed, *P<0.05; **P<0.01, compared to the TFAM-Apex2 GAL4 control).

We then analyzed the abundance of mtDNA and TFAM, as well as their association by confocal microscopy probing with antibodies against dsDNA and the flag-tag of TFAM (Fig. 3A). The mtDNA and TFAM-nucleoids appeared as bright spots under confocal microscopy imaging in the IFM of the control flies. The *mtRNAPol-*knockdown flies showed significant reductions of the spot intensities of mtDNA so as TFAM-Apex2 (Fig. 3A,B). However, the majority of mtDNA remains associated with TFAM, even though the total mtDNA content was reduced in the *mtRNAPol-*knockdown flies (Fig. 3C). On the other hand, a reduced fraction of TFAM remained associated with mtDNA (Fig. 3C). The data suggested that a reduction of TFAM was presented as the mtDNA-associated nucleoid form, given a reduced amount of mtDNA presents. Even though lower fluorescent signals of TFAM spots were observed, the western blot did not reflect the reduction of protein level in the knockdowns (Fig. S3c). Partly it may be resulted from the dispersed TFAM proteins throughout the mitochondrial matrix having lower fluorescent intensities that were filtered out by the threshold cutoff in the analysis.

Similar effects of the *mtTFB2*-knockdown on the mtDNA content and TFAM protein distribution were observed. The *mtTFB2*-knockdown flies showed a significant reduction of mtDNA and TFAM spot intensities (Fig. 3A,B). The majority of mtDNA associates with TFAM while a reduced fraction of TFAM presents as the mtDNA-associated form (Fig. 3A,C). Together, these results suggested that the reduced fraction of mtDNA-associated TFAM-nucleoid assemblies may have resulted from the reduction of mtDNA content by the *mtTFB2*-knockdown, in addition to the *mtRNAPol-*knockdown. On the contrary, the *mtTFB1-*knockdown had minimal effect on mtDNA content and the fraction of TFAM associated with mtDNA (Fig. 3A–C).

### The *mtRNAPol-* and *mtTFB2*- knockdown display reduced TFAM-nucleoid assemblies

**Fig. 4.**
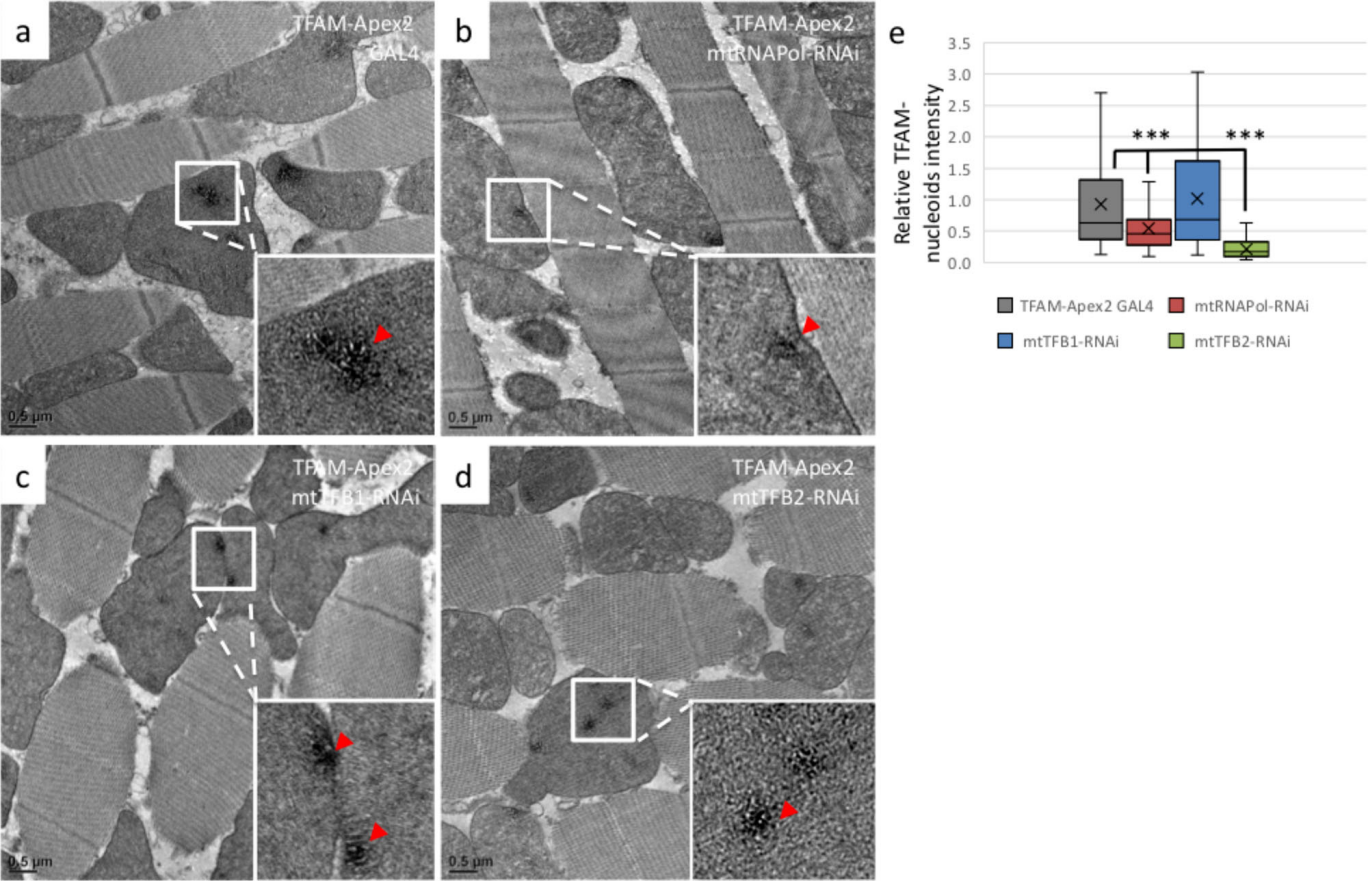
**The *mtRNAPol-* and *mtTFB2*- knockdown display reduced TFAM-nucleoid assemblies.** EM images of Apex2-staining in the IFM of TFAM-Apex2 GAL4 control flies (A) and flies with *mtRNAPol* (B), *mtTFB1* (C), and *mtTFB2* (D) knockdown. Red arrowheads: TFAM-Apex2 signals. (E) The analysis of the staining intensities of TFAM-Apex2 relative to the control is plotted in box-and-whisker graphs. The *t*-test was performed and statistically significant differences between indicated groups are marked with asterisks (*n*=47-114; ***P<0.01 compared to the TFAM-Apex2 GAL4 control).

We further analyzed the TFAM-nucleoid structure under *mtRNAPol-*, *mtTFB1-*, and *mtTFB2*- knockdown by TFAM-Apex2 staining for EM. The *mtRNAPol-* and *mtTFB2-*knockdown flies had significantly lower intensities of Apex2 staining (TFAM-nucleoids), with respective average intensities of 0.54 and 0.32 compared to the control flies (Fig. 4A–E). On the other hand, the TFAM-nucleoid organization was not altered significantly in the *TFB1-*knockdown flies, with an average intensity of 0.79 compared to the control flies (Fig. 4C,E). The Apex2-EM data suggested that mtNucleoids in the *mtRNAPol-* and *mtTFB2-*knockdown flies were formed from relatively low amounts of TFAM protein (Fig. 4A–E).

Our results suggest that altering mtDNA transcription by knocking down *mtRNAPol* and *mtTFB2* cross-regulates mtDNA replication to cause significant reductions in mtDNA content and TFAM-nucleoid assembly. mtDNA content appears to regulate TFAM-nucleoid assembly critically.

## DISCUSSION

mtDNA replication and transcription are dynamically regulated in response to cellular physiology (Barshad et al., 2018; Bogenhagen et al., 2008; Kang et al., 2018; Nicholls and Gustafsson, 2018). The higher order mtNucleoid structure is important for the maintenance of mtDNA stability and integrity, which are thought to participate in aging and various diseases (Kang et al., 2018; Nicholls and Gustafsson, 2018). In this study, we assessed how *in vivo* TFAM-nucleoid structure changes during the *Drosophila* adult lifespan and showed the smaller and more compact TFAM-nucleoids in aged flies. Furthermore, we revealed the cross-regulation of mtDNA transcription and replication in the *mtTFB2*-knockdown flies, in addition to the *mtRNAPol*-knockdown flies that alter TFAM-nucleoid packaging. The data suggested that mtDNA content critically regulates TFAM-nucleoid assembly.

The structure and function of mtNucleoids is an area of active research. A recent study identified 37 proteins that exist within mtNucleoids using Apex2-mediated proximity labeling (Han et al., 2017). Moreover, mtNucleoid packaging was characterized by super-resolution microscopy, EM, and correlative microscopy in mammalian cell cultures (Brown et al., 2011; Kopek et al., 2012; Kukat et al., 2011). The super-resolution microscopy studies revealed that mtNucleoids have a mean diameter of 100 nm (85×108×146 nm) and contain an average of 1.4 to 2.98 mtDNA copies; previous cellular studies had reported 2–8 nucleoids per mitochondrion (Brown et al., 2011; Kopek et al., 2012; Kukat et al., 2011). Here, we reveal the organization of mtNucleoids in *Drosophila* flight muscle tissue *in situ* under physiological conditions. mtNucleoids were probed at the ultrastructure level through the use of a newly generated TFAM-Apex2 fly model. During the active mitochondrial maturation that occurs just after the eclosion of adult flies (Jiang et al., 2020), TFAM-nucleoids appeared more dispersed in spatially less confined mitochondrial matrix compartments. As flies aged, the appearance of smaller and more compact TFAM-nucleoids was evident.

We have elucidated further that mtDNA transcription and replication are cross-regulated in the *mtTFB2*-knockdown flies, in addition to the *mtRNAPol-*knockdown flies. The reduction in mtDNA content resulted in a decreased fraction of TFAM associated with mtDNA that forms nucleoid packaging. mtRNAPol was shown to prime the lagging-strand mtDNA synthesis *in vitro* (Fuste et al., 2010; Wanrooij et al., 2008). mtRNAPol regulates the switch between replication primer formation and gene expression on mammalian mtDNA (Kuhl et al., 2016). Whether the cross-regulation of replication and transcription in the *mtTFB2*- knockdown involves mtRNAPol or proceeds through other mechanisms remains to be elucidated. Overall, our findings show the modulation of TFAM-nucleoid organization *in situ* under physiological aging of *Drosophila.* In addition, mtDNA transcription and replication are cross-regulated in *Drosophila* and alter the TFAM-nucleoid association and organization.

## MATERIALS AND METHODS

### Fly strains

*Drosophila* strains w1118 were used. The Apex2-Flag-tagged TFAM knock-in flies were generated by CRISPR/Cas9-mediated genome editing and homology-directed repair using a guide RNA(s) and dsDNA plasmid donor. The PBac system was used for genetic screening (Well Genetics). The construct design is as follows and the protein sequence after editing was shown in Fig. S1.

Guide RNA Primers: Sense oligo 5′-CTTCGCCAAAGCCCCGCAAGACGC;

Antisense oligo 5′-AAACCGCGTCTTGCGGGGCTTTGGC.

PAM mutation: GCCAAAGCCCCGCAAGACGC[TGG] CTG→CTC/ L→L.

Upstream Homology Arm: 1083 bp, −1086 to −4 nt relative to stop codon of TFAM.

Forward oligo 5′-TGCCAATCCCCAGATTACCAC;

Reverse oligo 5′-TATATCTTTGGAGGCGAGCGT.

Downstream Homology Arm: 1026 bp, +1 to +1028 nt relative to stop codon of TFAM.

Forward oligo 5′-TTGTAGCTGCTCGGCCCGC;

Reverse oligo 5′-AAATGATGCAGAAGTGGCT.

TFAM-APEX2 on *mtRNAPol-, mtTFB1-,* and *mtTFB2-RNAi* background was expressed in the IFM under the control of *Actin88F-GAL4* (Bloomington 38461). The *UAS-RNAi* lines used in the study were *P{TRiP.GLC01346}attP40* (for *mtRNAPol*; Bloomington 44419), *P{TRiP.JF02890}attP2* (for *mtTFB1*; Bloomington 28054), *P{TRiP.JF02381}attP2* (for *mtTFB2*; Bloomington 27055). The genotypes of the flies were as follows.

**Table:**
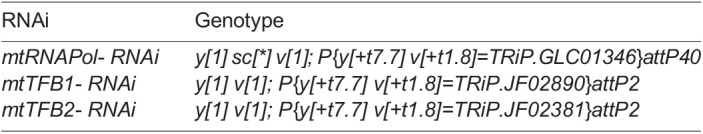


### Quantitative PCR

Fly DNA was extracted by homogenizing about 50 thoraxes in 200 μl of buffer containing 10 mM Tris (pH 8.0), 1 mM EDTA, 25 mM NaCl. Lysates were supplemented with 0.2 mg∕ml Protease K and incubated at 45°C for 30 min, followed by inactivation at 95°C for 5 min. The supernatant was collected after centrifugation. 100 ng of DNA were used in the qPCR reaction. mtDNA content was analyzed by qPCR using SYBR Green I Master and a LightCycler^®^ 480 (Roche). The relative mtDNA copy number was normalized to the Rp49 copy number, according to the calculated threshold cycle (Cp); the value was then normalized to day 1 samples. Each run was performed in triplicate; standard deviations of Cp were less than 0.5 cycles.

For the analysis of gene transcripts, fly RNA was extracted using RNeasy Mini Kit (Qiagen) and reverse-transcribed using a RevertAid First Strand cDNA Synthesis Kit (ThermoFisher Scientific). cDNA (100 ng) was used in the qPCR reaction. The relative mtDNA transcript levels were normalized to the RpL32 transcript level and subsequently normalized to wk1. Each run was performed in triplicate with a standard deviation of Cp less than 0.5. Statistical analyses were performed by the *F*-test and *t*-test functions in Excel. Specific primers used in the study were as follows.

**Table:**
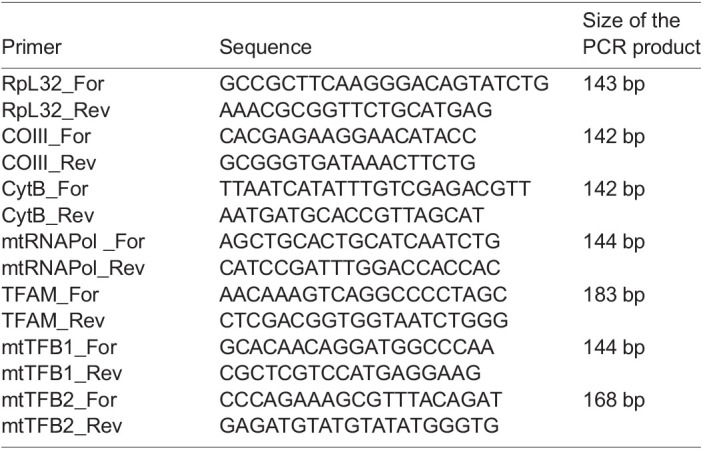


### Immunofluorescence staining

Fly thoraxes were cut in half in the fixation buffer containing 4% paraformaldehyde and 1% Triton X-100 in PBS and fixed for 20 min at RT without shaking. The specimens were washed with 0.1% Triton X-100 in PBS for 20 min at RT three times. After blocking with 5% normal goat serum (Jackson Immuno Research) in 0.1% Triton X-100 in PBS for 2 h at RT, the specimens were stained with primary antibodies, mouse anti-dsDNA (1:1000, abcam ab27156) and rabbit anti-Flag (1:1000, Invitrogen PA1-984B), in blocking buffer overnight at 4°C. After washing with 0.1% Triton X-100 in PBS for 20 min at RT three times, the specimens were stained with secondary antibodies (anti-mouse IgG Alexa-488, anti-rabbit IgG Alexa-594, 1:500, Jackson Immuno Research) and Alexa Fluor 647 Phalloidin (1:1000, Invitrogen A22287) in blocking buffer overnight at 4°C. After washing with 0.1% Triton X-100 in PBS for 20 min at RT three times, the specimens were mounted on the glass slides. Volumes of 84.2×84.2×5 μm^3^ were imaged with a LMS880 (Zeiss) confocal microscope.

The fluorescence intensities of mtDNA and TFAM, and their colocalization were analyzed using Imaris image analysis software. To analyze the fluorescence signal from mtDNA, the original dsDNA signals were filtered by masking the signal from nuclear DNA. Subsequently, the signals of mtDNA and TFAM were approximated as spots in Imaris. The colocalization of mtDNA and TFAM spots and the median intensities were plotted in box-and-whisker graphs. Statistical analyses were performed by the *F*-test and *t*-test functions in Excel.

### Western blot analysis

Fly thoraxes were homogenized in RIPA buffer containing protease inhibitors (cOmplete™, Roche) using a Dounce tissue grinder. Cellular debris was removed by centrifugation at 14,000× ***g*** for 20 min at 4°C. The supernatants were collected and the protein concentrations were determined by Pierce protein assay (Pierce 660 nm Protein Assay Reagent, ThermoFisher Scientific). Proteins were loaded at 20 μg/well for SDS-PAGE and western blot analysis.

Mouse anti-ATP5A (1:50,000, Abcam ab14748), mouse anti-Flag M2 (1 μg/ml, Sigma F3165), rabbit anti-alpha tubulin (1:10,000, Abcam ab18251), anti-mouse IgG-HRP (1:2000, Invitrogen 62-6520), and anti-rabbit IgG-HRP (1:5000, Abcam ab97051) antibodies were used in the study. For quantification, the densitometry signal for individual proteins was normalized to alpha-tubulin. The ratios were then normalized to the day 1 sample. Three independent samplings were analyzed and the mean±s.d. was plotted. Statistical analyses were performed by the *F*-test and *t*-test functions in Excel.

### Thin-section TEM for morphological observation

Flies were anesthetized on ice and embedded in 4% low melting agarose in 0.1 M phosphate buffer. Embedded flies were then sectioned at 100 μm with a vibrating blade microtome (Leica VT1200S) and fixed in 2% glutaraldehyde in a buffer containing 0.1 M sodium cacodylate with 2 mM CaCl_2_, pH 7 for 60 min, followed by five washes at 2 min each. The sections were post-fixed in 2% osmium tetroxide for 30 min, then washed five times for 2 min each, and incubated in 2% uranyl acetate overnight. After dehydration in solutions with ascending ethanol percentage, the specimens were infiltrated and embedded in Spurr's resin, then polymerized at 65°C for 16 h. The specimen blocks were trimmed and subjected to sectioning using an ultramicrotome. The sections were stained with 2% uranyl acetate for 10 min. Reynold's lead citrate was applied for 4 min, then samples were subjected to TEM inspection.

### Apex2 staining EM

The protocol was performed as previously described, with slight modifications (Hung et al., 2016). Vibratome sections of fly tissue were fixed in 2% glutaraldehyde in buffers containing 0.1 M sodium cacodylate with 2 mM CaCl_2_, pH 7. Residual glutaraldehyde was washed off (five washes for 2 min each) and quenched with 20 mM glycine followed by five more washes. The specimens were subsequently stained with SIGMA *FAST*™ DAB (3,3′-diaminobenzidine tetrahydrochloride) with Metal Enhancer Tablets (Sigma-Aldrich) for 20 min, washed in the buffer (10 min each, five times), and stained with 1% osmium tetroxide for 30 min. After washing with ddH2O (10 min each, three times), the specimens were stained with 1% uranyl acetate overnight. The specimens were then further dehydrated and embedded in resin for thin-sectioning and TEM observation.

TFAM-Apex2 staining signals were analyzed using Fiji ImageJ software. First, Gaussian Blur filters were applied to the TEM images, and then, the threshold was adjusted to isolate TFAM-Apex2 signals. The intensities and area of individual TFAM-nucleoids were plotted in box-and-whisker graphs. Statistical analyses were performed by the *F*-test and *t*-test functions in Excel.

### ATP measurements

Thoraxes of ten flies were homogenized in 200 μl ice-cold buffer (6 M guanidine-HCl, 100 mM Tris, and 4 mM EDTA, pH 7.8) using a Dounce tissue grinder. Supernatants were collected after centrifugation at 16,000× ***g****.* The protein concentrations were determined by Pierce protein assay (Pierce 660 nm Protein Assay Reagent, ThermoFisher Scientific). ATP content was measured by the CellTiter-Glo^®^ Luminescent Cell Viability Assay (Promega); 100 μl of samples at 20 pg/μl per well were used. The ATP content was normalized to the control samples. Three independent samplings were analyzed and the mean±s.d. was plotted. Statistical analyses were performed by the *F*-test and *t*-test functions in Excel.

### Climbing assay

Flies were transferred to new culture tubes one day before the analysis. On the day of analysis, a hundred flies were transferred to a 100-ml graduated cylinder and knocked down to the bottom of the cylinder. Then the flies were video recorded climbing up to the 100 ml mark on the cylinder (about 18 cm height). The numbers of flies climbing to the target line were recorded in 10 s bins for 120 s and plotted in triplicate. Three independent samplings were analyzed and the mean±s.d. was plotted. Statistical analyses were performed by the *F*-test and *t*-test functions in Excel.

## Supplementary Material

Supplementary information
