# Supplementary material for: Modulation of mitochondrial nucleoid structure during aging and by mtDNA content in Drosophila
Source: Biol Open. 2021 Jun 28;10(6):bio058553. doi: 10.1242/bio.058553 (PMC8380045; doi:10.1242/bio.058553)
Supplement: Supplementary information [file biolopen-10-058553-s1.pdf]

Biology Open (2021): doi:10.1242/bio.058553 Supplementary information

Fig S1

The last CDS exon of TFAM is shown here.
NNNNN: TFAM
NNNNN: 10aa linker
NNNNN: APEX2
NNNNN: Flag

KAISVWEEKMetIRLGHIDVVRHGNLIDPPEPKPRKTLASKDIKGSGSTSGSGGKSYPTVSADYQDAVEKAK
KKLRGFIAEKRCAPLMetLRLAFHSAGTFDKGTKTGGPFGTIKHPAELAHSANNGLDIAVRLLEPLKAEFPILS
YADFYQLAGVVAVEVTGGPKVPFHPGREDKPEPPPEGRLPDPTKGSDHLRDVFGKAMetGLTDQDIVALS
GGHTIGAAHKERSGFEGPWTSNPLIFDNSYFTELLSGEKEGLLQLPSDKALLSDPVFRPLVDKYAADEDAFF
ADYAEAHQKLSELGFADADYKDDDDKStop

Fig S1. Construct design of the TFAM-Apex2 knock-in fly

The protein sequence of the last CDS exon of TFAM and the Apex2-Flag knock-in.

Biology Open • Supplementary information

Fig S2

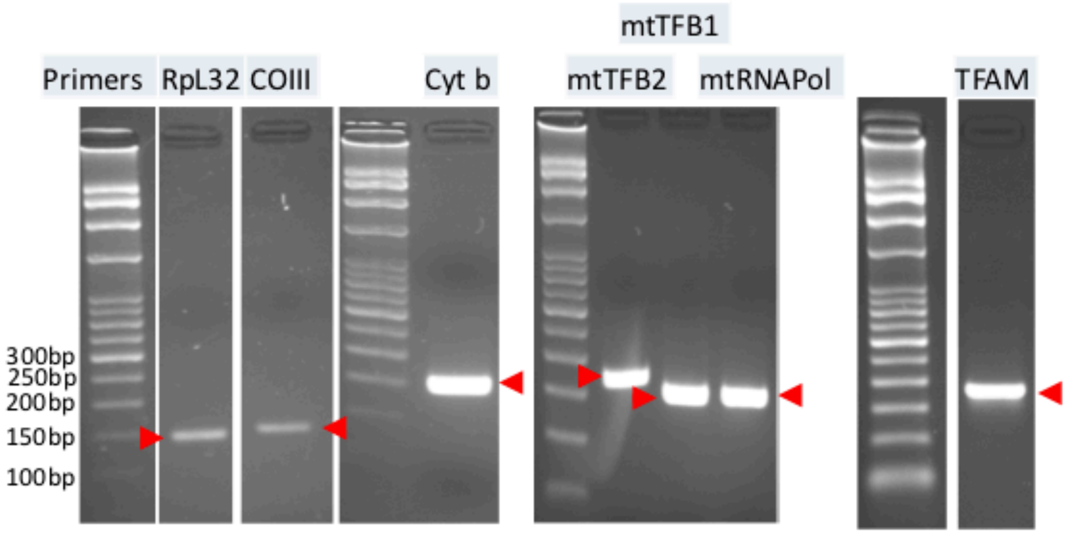

Fig S2. Gel electrophoresis of PCR reactions

Gel electrophoresis of qPCR reactions. Probes for RpL32 and COIII were used to amplify 100 ng DNA templates, and Cyt b, mtRNApol, TFAM, mtTFB1, and mtTFB2 specific primers were used with 100 ng cDNA. Red arrowheads indicate specific products of the predicted size.

Fig S3

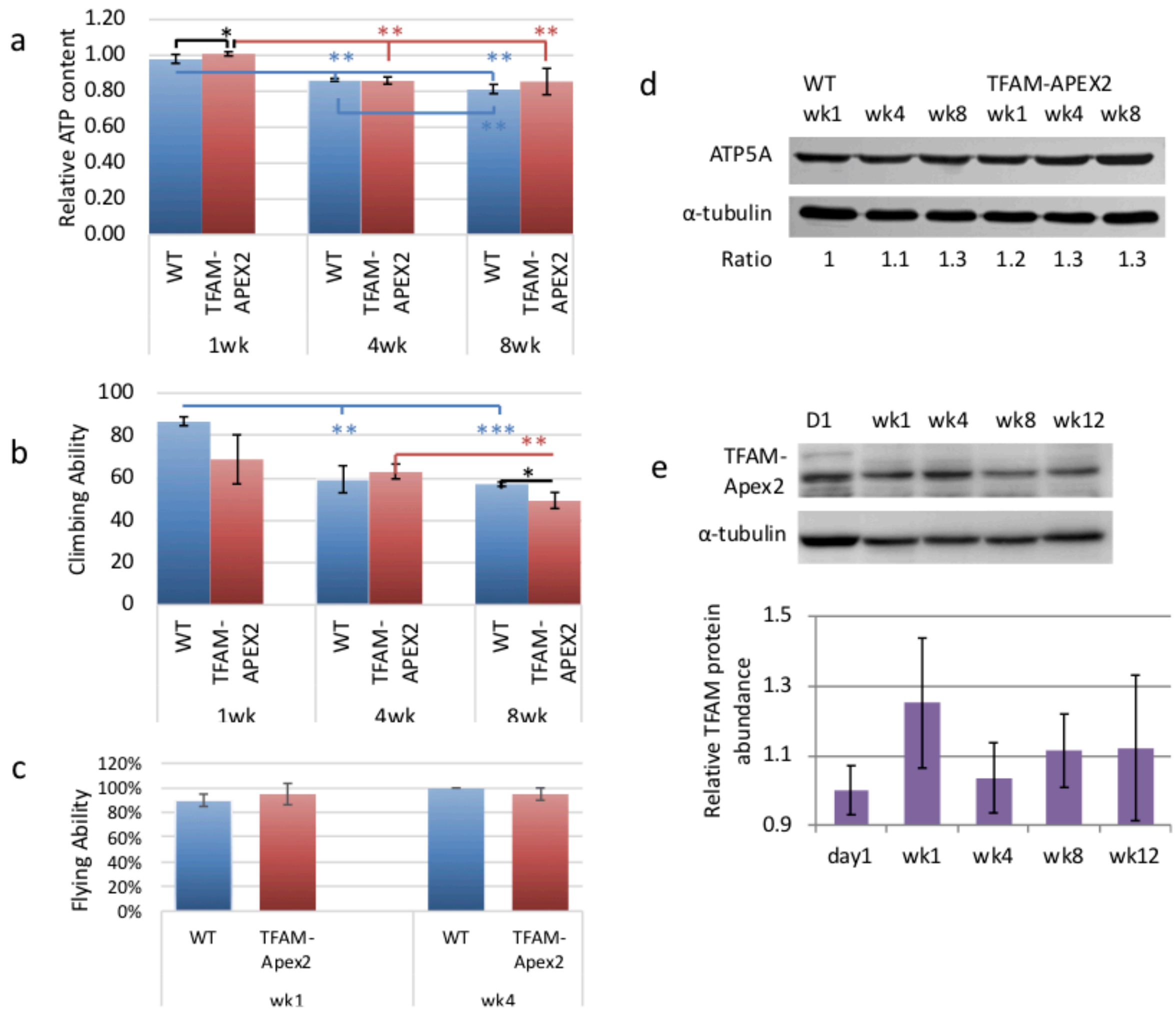

Fig S3. Characterization of TFAM-Apex2 flies

(a) ATP content of wild-type and TFAM-Apex2 flies at weeks 1, 4, and 8 post-eclosion was measured and normalized to wild types at week 1. The mean +/- s.d. of the triplicates was plotted. (b) The climbing abilities of wild-type and TFAM-Apex2 flies at weeks 1, 4, and 8 were measured. N=100 and the mean +/- s.d. of the triplicates was plotted. The t-test was performed and statistically significant differences between indicated groups are marked with asterisks (*, $p < 0.05$; **, $p < 0.01$; ***, $p < 0.001$). (c) The flying abilities of wild-type and TFAM-Apex2 flies at weeks 1 and 4 were measured. N=20 and the mean +/- s.d. of the triplicates was plotted. (d) ATP5A protein levels of wild-type and TFAM-Apex2 flies at weeks 1, 4, and 8 were analyzed by western blot using the antibody against ATP5A. (e) TFAM protein expression of TFAM-Apex2 flies detected by western-blot. The mean +/- s.d. of the triplicates was plotted and the t-test was performed. Quantification of the triplicates showed similar TFAM abundance in flies of different ages.

Biology Open • Supplementary information

Biology Open (2021): doi:10.1242/bio.058553 Supplementary information

Fig S4

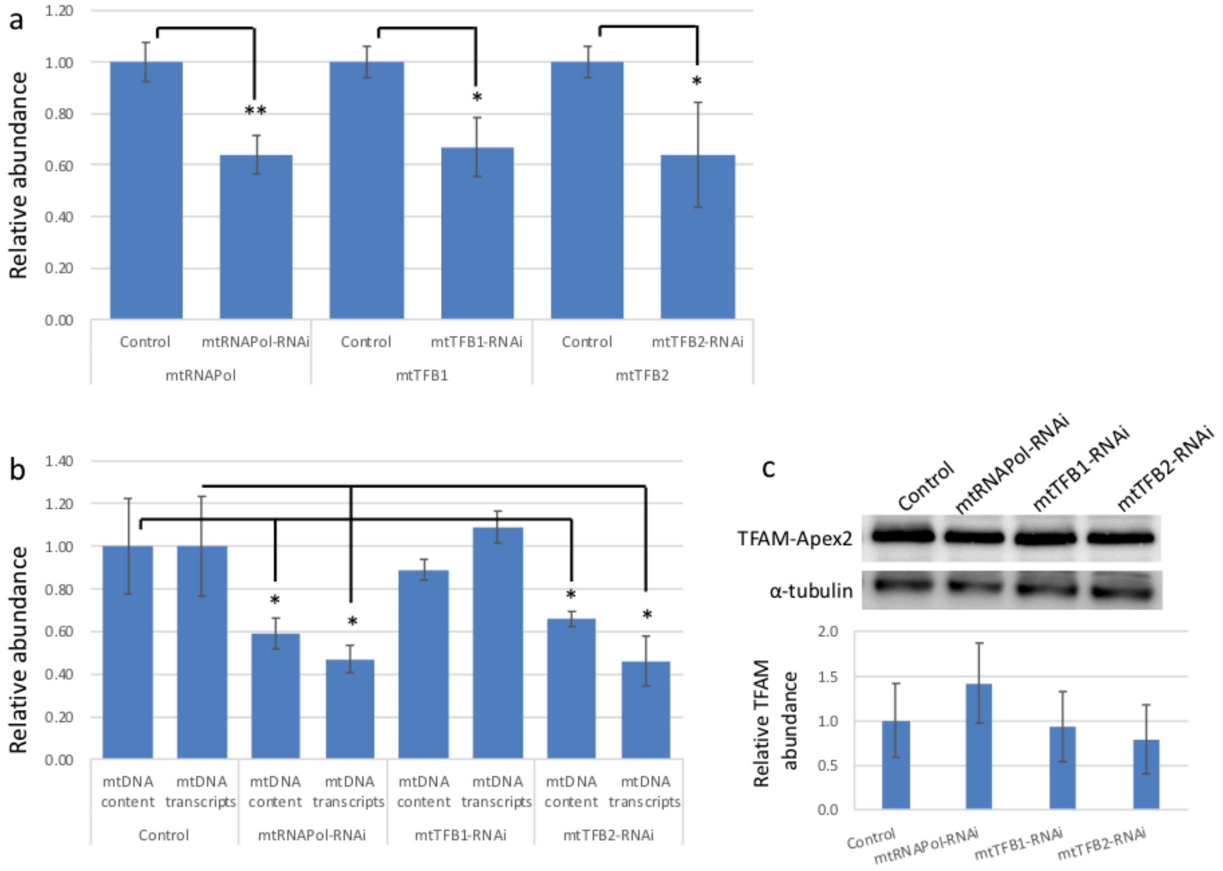

Fig S4. Characterization of the *mtRNAPol*-, *mtTFB1*-, and *mtTFB2*-knockdown flies

(a) The gene transcripts of *mtRNAPol*-, *mtTFB1*-, and *mtTFB2*- knockdown flies were analyzed by qPCR. (b) The mtDNA content and transcripts of *mtRNAPol*, *mtTFB1*, and *mtTFB2*- knockdown flies were analyzed by qPCR and compared to TFAM-Apex GAL4 controls. The mean +/- s.d. of the triplicates was plotted. The t-test was performed and statistically significant differences between indicated groups are marked with asterisks (*, $p < 0.05$; **, $p < 0.01$). (c) The TFAM expression levels in the *mtRNAPol*-, *mtTFB1*-, and *mtTFB2*-knockdown flies were analyzed by western blot and quantified with triplicates. The mean +/- s.d. of the triplicates was plotted and the t-test was performed.
